# Supplementary material for: Chromothripsis during telomere crisis is independent of NHEJ, and consistent with a replicative origin
Source: Genome Res. 2019 May;29(5):737–49. doi: 10.1101/gr.240705.118 (PMC6499312; doi:10.1101/gr.240705.118)
Supplement: Supplemental Material [file supp_gr.240705.118_Supplemental_file_1.zip › contigs/annotated_contigs/DB49/contig.3.DB49_length_386_mean_cov_10.0984455959.docx]

**DB49_length_386_mean_cov_10.0984455959**

```
AACTACACAACATCATAACACTACGACTCCAAGCACACACA|AAAACAAAACAAAAAAACAATGCCGGGCAGTGACTGTCACCTGTAAT
                                          >chr19:53278504-53278757 + E=6e-141
CCCAGCACTTTGGGAGACCAAGACGGGCAGATAATGTGGTCAAGAGATTGAGACCATCCTGGTCAACATGGCAAAACCATGTCTCTACT

AAAAATACAAAAATTAGCTGGGTGTCGTGGTGCAGGCCTGTACTCCCAGCTACTCAAGAGGCTGAGGCAGGAGGATCGCATGAATCCGA

GAGGTGGAGGTTACAGTGAGCCAAGATC|ATTC|CATTGCACTCCACCTTTGGGGACTGAGCGAGACTCCATCTTAAAATA|AGTA|AA
                                 >chr19:53006157-53006204 - E=9e-11                 >
TAAATAGATAGAGAGATACATATATACATACA|TACchr22:36613242-36613276 - E=6e-06
```
